# Fidelity Scale: From Black Box to Holy Grail

**DOI:** 10.1007/s10488-020-01057-8

**Published:** 2020-06-04

**Authors:** Jaap van Weeghel

**Affiliations:** 1 Tranzo Scientific Center for Care and Welfare, Department of Social and Behavioral Sciences, Tilburg University, Tilburg, The Netherlands; 2 Parnassia Psychiatric Institute, The Hague, The Netherlands; 3 Phrenos Center of Expertise, Da Costakade 45, 3521 VS Utrecht, The Netherlands

## Abstract

Fidelity scales are indispensable in the pursuit of evidence-based mental healthcare. Without fidelity checks, treatment remains a mysterious black box. The aim of this article is to comment on the studies in this special section, and to discuss some general issues with regard to fidelity assessment. Despite all of their supposed benefits, resistance to fidelity scales persists among mental health practitioners. One way to overcome this resistance is to conduct fidelity assessments in the context of a well-guided learning community. The predictive validity of fidelity scales is considered the single most valuable attribute of these instruments. Research on predictive validity requires large sample sizes, which is difficult to achieve. It should nevertheless not prevent us from rigorously searching for this Holy Grail of fidelity assessment. In addition, fidelity assessment should be placed in a broader perspective. The quality of care for people with severe mental illness cannot be assessed conclusively according to the extent to which separate interventions have been applied with good fidelity. These individuals need access to high-quality treatment and support systems within the community, which can enable them to live their lives as valued citizens. In conclusion, fidelity assessment, both at the level of interventions and systems, contributes to a highly desirable transparency in practice variations within the field of mental healthcare.

## Introduction

Treatment fidelity is indispensable in the pursuit of evidence-based mental healthcare. In most cases, fidelity scales reflect the consensus reached amongst experts with regard to the characteristics and requirements of specific interventions. Fidelity scales also facilitate research aimed at identifying, measuring and improving the components and effects of interventions (Bond and Drake 2019). Without fidelity checks, treatment outcomes are subject to either overestimation or underestimation (Moncher and Prinz 1991), and it is not possible to distinguish failure of the intervention from failure to implement the intervention (Mowbray et al. 2003). Without fidelity checks, treatment remains a mysterious black box. We do not know exactly what an intervention is, how to implement it, with which level of quality it is delivered or how it differs from other interventions (Bond and Drake 2019).

During the first decade of this century, Bob Drake, Greg McHugo, Will Torrey and colleagues conducted the National Implementing Evidence-Based Practices Project in 53 sites in 8 states of the United States (Drake et al. 2001). Fidelity scales were administered for Assertive Community Treatment (ACT), Individual Placement and Support (IPS), Family Psychoeducation (FPE), Illness Management and Recovery (IMR), and Integrated Dual Diagnosis Treatment (IDDT) (McHugo et al. 2007). This ground-breaking project gave momentum to fidelity assessments in interventions for people with severe mental illness (SMI). The contributions to this issue provide evidence that fidelity assessment has also taken off in Europe. This is the case in Norway, as well as in the Netherlands, where various fidelity scales are being used on a regular basis for purposes of both research and implementation (e.g. Sanches et al. 2018; van Vugt et al. 2011; Van Weeghel et al. 2020). Fidelity assessment has become a global endeavour, as particularly reflected in treatment programmes for people with first-episode psychosis. To date, no fewer than six fidelity scales for such programmes are in use in different parts of the world. Attempts are being made to achieve a generally accepted synthesis of these scales (Addington et al. 2018).

In this article, three general issues are discussed: overcoming resistance to fidelity scales, the use of fidelity scales in mental healthcare systems and the importance of predictive validity. But I start with some specific comments on the Norwegian studies in this special section.

## Fidelity Scales Examined in Norway

Fidelity scales should demonstrate interrater reliability, discriminative validity, sensitivity to change, predictive validity and usability, in addition to having empirical benchmarks. Many fidelity scales have yet to satisfy these criteria (Bond and Drake 2019). One of the greatest merits of the project conducted by the research team from Norway is that they evaluated psychometric properties of no less than five fidelity scales. However, their studies also give rise to some important questions.

First of all, quality of care is to a large extent in the eye of the beholder (van Weeghel et al. 2011). One limitation of the Norwegian fidelity assessments is therefore that they do not address the views and experiences of service users and their families. They also do not involve any observations of the interventions in practice. Although these omissions are mentioned as major limitations in all five articles, it remains unclear why the researchers were willing to accept these limitations, given that they are essential elements of any fidelity assessment (Bond and Drake 2019).

Another observation is that, on the whole, the psychometric properties of the scales examined were found to be satisfactory. However, the fidelity scales that have been around for some time—addressing Family Psychoeducation (FPE; Joa et al. 2020), Illness Management and Recovery (IMR; Egeland et al. 2019) and the General Organizational Index (GOI; Heiervang et al. 2020)—overall had slightly better results than those that have been developed more recently for physical healthcare (Ruud et al. 2020a) and antipsychotic medication management (Ruud et al. 2020b). This is probably due, at least in part, to the fact that the earlier scales have a longer history of testing and improvement. Alternatively, the finding could also be related to the type of interventions involved and the professional groups implementing them.

With regard to the more recent scales, the assessed practices were more successful in establishing policies specifying standards for physical healthcare than they were in implementing these policies in daily practice (Ruud et al. 2020a). However, the reverse was true with regard to the scale for antipsychotic medication management: the implementation of prescriber fidelity was slightly more successful than was the implementation of policy fidelity (Ruud et al. 2020b). Another intriguing question concerns why the implementation of IMR (Egeland et al. 2019) proceeded so well and was so much more successful than was the case for the other interventions. This might have been due to the characteristics of the intervention, the expertise and motivation of the practitioners involved, or it might have had more to do with organizational conditions. Finally, it is interesting to note that the mean GOI score at baseline was practically ‘1’ (Heiervang et al. 2020). This result seems unlikely as it suggests that individualization of treatment and quality improvement were completely non-existent in the participating organisations at the start of the project.

External commentators can only guess about the explanations for these intriguing results. It would thus be enlightening if this series of articles is followed by an article describing the background to and the rationale for the project as a whole, and reflecting on the research results from an insider’s perspective.

## Overcoming Resistance to Fidelity Measures

In this series of articles, it has been repeatedly stated that regular fidelity monitoring is needed in order to achieve permanent quality improvement, but that such monitoring is considered difficult to implement. Regular assessments may be relevant to many stakeholders, including funding bodies (e.g. to provide reassurance that their investments are reaching the expected population), service managers (e.g. to improve the distribution of resources), clinicians (e.g. to identify strengths and areas for improvement), service users (e.g. to provide evidence of the desired outcomes) and institutions (e.g. to establish accreditation and licensing criteria) (Alvarez Monjarás 2019). The likelihood that regular monitoring will get off the ground and the results that it will yield is determined largely by the priorities of these stakeholders.

Despite all of their supposed benefits, resistance to fidelity scales persists (Egeland 2018; Alvarez Monjarás 2019). In the Netherlands, a significant number of mental health practitioners fear that fidelity scales, or even evidence-based practices in general, will lead to an undesirable lack of variety in treatment practice, while frustrating the creativity and individuality of professionals. These opponents believe that clients ‘should not be stuffed into the moulds of evidence-based practices’, as every client is unique and suffers from an incomparable tangle of problems. In addition, the social contexts in which clients lead their lives and experience problems varies from one individual to another. What is therefore needed is an individual, customised approach. The use of fidelity scales is regarded by some as yet another attempt by managers to further control the actions of skilled, yet idiosyncratic professionals, thereby also limiting the treatment options of service users.

This view is held by some mental healthcare practitioners, but it is even more common amongst professionals in the social services. Most of these practitioners have no affinity with evidence-based practices, and they sometimes vehemently criticise them. This is a challenge: our complex ‘multi services and multi-budget world’ (McDaid and Thornicroft 2005) requires mental healthcare practitioners and professionals in the social services to engage in extensive collaboration in order to provide mutual clients with the integrated care and support they need.

The justification of such criticism is debatable. In the development of any fidelity scale, it is important to find the proper balance between precisely formulated prescriptions and sufficient leeway for professional discretion and individual variation. This leads to a paradox in which freedom of choice, shared decision-making and individualisation are mandatory and standardised elements in most of the fidelity scales that are currently in use. Even if a particular fidelity scale lacks one or more of these elements, it is always possible to use the GOI—a meta-fidelity scale that assesses the level of individualisation in the delivery of care. More specifically, it assesses the extent to which interventions are customised to meet the needs values, goals and choices of each client.

The distinction between formative and summative assessments (Harlen and James 2006) may be relevant in this context. Formative assessments are typically conducted during the development or improvement of an intervention. Such assessments are intended to provide as much feedback as possible, which is subsequently used to provide practitioners with specific advice before, during and at the conclusion of the implementation process with regard to filling specific gaps. Summative assessments involve making judgments about the efficacy of an intervention upon its conclusion. A summative assessment takes stock of the development process: Are we meeting the standards associated with our goal? More formal types of measurement are indicated for summative assessments, with the relationship between practitioner and assessor shifting into the background.

Most practitioners are likely to be more motivated when the regular monitoring of fidelity consists of formative assessments. Such monitoring will probably yield the best results when the assessments are conducted in the context of a well-guided learning community. Such communities offer forms of collaboration and friendly competition between participants, in addition to providing a comprehensive toolkit (e.g. a manual, a supervision guide, courses and other development opportunities). One successful example is the international learning collaborative for IPS (Becker et al. 2014; Bond et al. 2016).

## Fidelity Assessment of Mental Healthcare Service Systems

In addition to severe mental (and, in many cases, physical) health problems, people with SMI experience social disadvantages and unmet needs in other life domains. This implies that ‘one-issue’ interventions (e.g. medication management and family psychoeducation) should be supplemented with ‘multi-component’ interventions (e.g. ACT and IDDT), in which different services, treatment approaches, agencies and disciplines are involved and interact with each other to address the diverse range of client needs (Alvarez Monjarás 2019). Developing fidelity scales can be particularly challenging in interventions for people with SMI, given the large number of contextual, organisational and service-level components (Wheeler et al. 2015). The standardised methodology for the development and validation of such fidelity scales outlined by Bond and Drake (2019) thus constitutes a major advance.

It is important to note, however, that the quality of care for people with SMI cannot be assessed conclusively according to the extent to which a number of separate interventions have been applied with good fidelity. These individuals need access to high-quality treatment and support systems within the community, which can enable them to live their lives as valued citizens. Even multi-component interventions are not sufficient to build a comprehensive service system on a local or regional scale. These interventions should be embedded within evidence-based environments, including systems and policies that contribute to the effectiveness of interventions and the recovery options of clients, along with sufficient financial resources, non-stigmatising social environments and social support programmes (Scheyett et al. 2006).

The past half century has witnessed the development of various models of community mental healthcare. In the 1970s, the concept of Community Support Systems (CSS) was developed in the United States, comprising 12 essential components needed to provide adequate services and support to people with SMI, ranging from mental healthcare treatment to rehabilitation services, and from outreach practice to system coordination (Anthony and Blanch 1989). Such support systems can be outlined from the client’s perspective using the Framework for Support (Carling 1995), which includes self-help, support from family, friends and peers, services by generic organisations and specialised mental healthcare services. The balanced care model developed by Thornicroft and Tansella (2013) is another example of a comprehensive multi-level system.

At the beginning of this millennium, regional care systems for people with schizophrenia and other psychotic disorders were developed in the Netherlands, using the multidisciplinary guidelines for schizophrenia as the main starting point. These care systems have been evaluated from multiple perspectives (i.e. clinicians, clients, families and community organisations), using an instrument known as QUARTS (Quality Assessment of Regional Systems for Schizophrenia). The QUARTS instrument can be regarded as a fidelity scale for assessing the availability and quality of all key elements of a comprehensive service system. Clinicians and other stakeholders have perceived the QUARTS instrument as helpful for the monitoring and development of services (Van Weeghel et al. 2011).

More recently, discussions among European mental health practitioners, peer experts and researchers have resulted in an overview of essential notions for a national, regional and local model of integrated mental healthcare. Six principles for high-quality community-based mental healthcare have been elaborated in a consensus paper: protect human rights; focus on public health; support service users in their recovery journeys; use effective interventions based on evidence and client goals; promote a wide network of support within the community; and use peer expertise in the design and delivery of services (Pieters et al. 2017; Keet et al. 2019). Mental healthcare organisations across Europe have expressed the desire to learn from each other by talking to and visiting the services of colleagues from institutions in different countries. The purpose of such site visits is to allow practitioners from different services to learn from each other with regard to the ways in which they bring the principles of good community mental healthcare into practice. A manual was developed to facilitate these mutual visits. One important aspect identified is that the visits should be non-judgemental and explicitly aimed at achieving a respectful and mutual exchange on an equal level.

## Predictive Validity: The Quest for the Holy Grail

Although fidelity scales can be used to show that a programme is delivering care as planned and at a certain level of quality, outcome-assessment measures are required in order to demonstrate their impact on the lives of service users (Hermann 2002). Important examples of meaningful outcomes include managing symptoms, staying out of hospital, living in a safe residence, having friends and family, and maintaining a satisfying job. Such measures reflect the predictive validity of fidelity scales, the single most valuable attribute of these instruments (Bond and Drake 2019). One common method for establishing predictive validity involves examining correlations between programme fidelity and the mean level of client outcomes at the programme level. The papers on the Norwegian studies also do not provide additional evidence about the predictive validity of the fidelity scales that they examine. As I understand from the principal investigator (T. Ruud) data on this were collected, but these results will be reported in one of the other papers from the study.

Bond and Drake (2019) report that fidelity scales are now being used in routine practice settings throughout the United States and many other countries, for the simple reason that higher fidelity to an EBP predicts better outcomes. Although this might be true in a general sense (Durlak and DuPre 2008), this situation also poses a risk that professionals will refer to this general knowledge and refrain from conducting targeted research into the predictive validity of specific fidelity measures. With regard to the treatment of people with SMI, evidence of predictive validity is still limited to the fidelity scales for ACT (Bond and Salyers 2004) and IPS (Bond et al 2012). Research on predictive validity requires large sample sizes, which is a ‘thorny issue’ (Bond and Drake 2019). It should nevertheless not prevent us from rigorously searching for this Holy Grail of fidelity assessment.

## Concluding Remarks

Placing fidelity assessment in a broader perspective, it is important to consider how much ‘practice variation’ is considered acceptable within a given mental health system (Coldefy et al. 2015). To what extent can mental healthcare organisations differ in the frequency and manner in which evidence-based treatment is provided to clients with similar health problems or social problems? Practice variation may be acceptable or desirable, for reasons including demonstrable differences in clients (or client populations) and their preferences, leeway in the interpretation and application of multidisciplinary guidelines, and the division of tasks across various mental healthcare organisations.

Fidelity assessment at the level of interventions and systems contributes to a highly desirable transparency in practice variations within the field of mental healthcare. What is at stake in this regard are the values of offering appropriate care and the accessibility of effective interventions to every citizen who needs them.
